# Complementary effects of extracellular nucleotides and platelet-derived extracts on angiogenesis of *vasa vasorum *endothelial cells *in vitro *and subcutaneous Matrigel plugs *in vivo*

**DOI:** 10.1186/2045-824X-3-4

**Published:** 2011-02-02

**Authors:** Mark Roedersheimer, Hala Nijmeh, Nana Burns, Asya A Sidiakova, Kurt R Stenmark, Evgenia V Gerasimovskaya

**Affiliations:** 1 Department of Surgery, University of Colorado Denver, 12700 East 19th Ave, Aurora, CO 80045, USA; 2 Department of Pediatrics, University of Colorado Denver, 12700 East 19th Ave, Aurora, CO 80045, USA

## Abstract

**Background:**

Platelets contribute to vascular homeostasis and angiogenesis through the release of multiple growth factors, cytokines and nucleotides, such as ATP and ADP. Recent reports have demonstrated a marked growth-promoting effect of total platelet extracts and selected platelet growth factors on therapeutic angiogenesis. However, since endogenous adenine nucleotides are rapidly degraded during the platelet isolation and storage, we examined whether supplementing a platelet-derived extract with exogenous adenine nucleotides would augment their pro-angiogenic effects.

**Methods:**

Pulmonary artery *vasa vasorum *endothelial cells (VVEC) were used to examine the effects of dialyzed platelet-derived soluble extracts and extracellular adenine nucleotides on proliferation, migration and tube formation. In addition, an *in vivo *Matrigel plug assay was used to examine the effects of platelet extracts and adenine nucleotides on neovascularization of plugs subcutaneously placed in 50 ICR mice. The number of vascular structures in Matrigel plugs were evaluated by histological and statistical methods.

**Results:**

Platelet extracts (6.4-64 μg/ml) significantly induced DNA synthesis and at a concentration of 64 μg/ml had a biphasic effect on VVEC proliferation (an increase at 48 hrs followed by a decrease at 60 hrs). Stimulation of VVEC with platelet extracts also significantly (up to several-fold) increased cell migration and tube formation on Matrigel. Stimulation of VVEC with extracellular ATP (100 μM) dramatically (up to ten-fold) increased migration and tube formation on Matrigel; however, no significant effects on cell proliferation were observed. We also found that ATP moderately diminished platelet extract-induced VVEC proliferation (48 hrs) and migration, but potentiated tube formation. Neither ATP, or a mixture of non-hydrolyzable nucleotides (ATPγS, ADPβS, MeSATP, MeSADP) induced vascularization of Matrigel plugs subcutaneously injected in mice, however, the combination of these nucleotides with platelet extracts dramatically increased the number of functional capillaries in the Matrigel plugs.

**Conclusion:**

Data from this study suggest that platelet-derived growth factors and extracellular nucleotides represent important regulatory signals for angiogenesis. Supplementation of platelet extracts with exogenous adenine nucleotides may reveal new possibilities for therapeutic angiogenesis and tissue regeneration approaches.

## Background

Angiogenesis is a fundamentally important process occurring under both physiological and pathological conditions, including embryonic development, tumor progression, and tissue regeneration. Regulation of angiogenesis requires a balance between multiple pro- and anti-angiogenic factors and involves interaction of the vascular wall and circulating blood cells [1-3]. Platelets, in addition to their role in hemostasis, can play a critical role in the modulation of angiogenesis with the capacity to release both pro- and anti-angiogenic factors [4,5]. In fact, platelet-rich plasma ("PRP") is currently used in a variety of settings to promote wound healing. However, the bioactivity, and optimal application of complex mixtures isolated from platelets, analogous to the materials currently widely used under the term "PRP", are not entirely understood. A better understanding will lead to enhanced efficacy in these applications.

Pro-angiogenic effects of platelet-derived soluble growth factors or platelet-derived microparticles have been demonstrated by several approaches including the rat aortic ring assay, subcutaneous agarose implants in mice [6], *in vitro *Matrigel tube formation and migration assay [7], electron microscopy, and intravital analysis of platelet in blood vessels [7,8]. Some studies demonstrated, that the majority of endothelial cell chemoattractant activity was generated during blood clotting, suggesting that platelet-released soluble factors, including lipid mediators are positive regulators of angiogenesis [9,10]. Whole platelets can also induce angiogenesis in the mouse matrigel plug assay, and this effect was shown to be dependent on multiple growth factors secreted by platelets [11]. It has been demonstrated that the presence of platelets not only stimulates angiogenic vessel growth but also plays a critical role in preventing hemorrhage from the angiogenic vessels [12]. Platelets have also been observed to contribute to tumor angiogenesis. Platelets of animals bearing malignant tumors actively sequester angiogenic mediators (VEGD, PDGF, bFGF) against a steep gradient from the serum, and therefore the levels of angiogenic mediators in the platelets can reflect the presence of an occult tumor while serum levels do not [5].

In addition to growth factors and metalloproteinases, platelet pro-angiogenic products also include lysophospholipids, such as sphingosine 1-phosphate (SP-1), lysophosphatidic acid (LPA) and possibly other butanol-soluble lipids [7,9]. Recent fascinating studies of Pula et al, demonstrated pro-angiogenic effects of thymidine phosphorylase [13] and platelet-derived deoxyribose-1-phosphate [14]. On the other hand, it is known that platelet activating factor-4, thrombospondin-1, plasminogen activator inhibitor-1, α_2_-macroglobulin, angiostatin and kinostatin exhibit anti-angiogenic effects [15,16]. Platelets contain three types of secretory vesicles: dense granules, α-granules, and lysosomes [17]. Pro- and antiangiogenic factors are stored in separate alpha-granule populations within platelets which can be released selectively in response to stimulation of specific platelet receptors [16]. This segregation may allow sequential release leading to safe and effective initiation and later suppression of regenerative (proliferative and migratory) activity during wound healing and tissue regeneration. Although single growth factors, such as VEGF or bFGF have been used to stimulate clinically relevant angiogenic responses, they failed to do so in isolation from other components present in natural platelet mixtures [18]. Alone, though very effective at stimulating proliferation and migration of endothelial cells in culture, these factors *in vivo *produced only weak and leaky immature blood vessels [19].

Extracellular purines and pyrimidines have long been known as regulators of vascular tone and permeability. Moreover, recent studies in different endothelial cell models have implicated the extracellular nucleotides in the regulation of angiogenesis [20-23]. Platelets are an abundant source of endogenous ATP and ADP, which are released from dense granules in response to platelet activation with thrombin, collagen, thromboxane or ADP itself. Thus, extracellular ATP and ADP may regulate vascular inflammation and thrombosis [24-26], as autocrine/paracrine signaling molecules. Both platelets and endothelial cells express P2 purinergic receptors responsive to extracellular nucleotides [27-29]. Extracellular ADP stimulates platelet aggregation via the activation of P2Y1 and P2Y12 receptors, coupled to phospholipase C and adenylate cyclase/cAMP pathways, respectively. In turn, extracellular ATP and adenosine antagonize the platelet aggregation response. Importantly, a recent study demonstrated that P2Y-mediated platelet activation resulted in release of VEGF and endostatin [30], indicating an involvement of platelets in purinergic regulation of angiogenesis.

As platelets are important to the angiogenic process, delivery of intact and "healthy" platelets (or their overall constituents) to a site of neovascularization would probably be ideal for therapeutic angiogenesis. It has been reported that liquid preserved platelet releasates retain the activity of endogenous growth factors [31]. However, how to consistently isolate intact and stable (unactivated) platelet concentrates from the bulk plasma environment remains controversial. Most recent methods of platelet extract preparation involve the separation of a fraction abundant in high molecular weight proteins from low molecular weight soluble components of total platelet extracts [11,32,33]. Recently, we have optimized a process for recovery of platelet protein mixtures, including their separation from the bulk serum proteins, and stabilization for long-term storage. The procedures of platelet extract preparation we have explored involve a dialysis step, thus the contribution of low molecular weight components, such as nucleotides, peptides, and other small molecules, are likely being lost using this approach. This could lead to a reduction in the angiogenic efficacy in these platelet extracts. In this study we tested the possibility that supplementation of platelet extracts with exogenous adenine nucleotides may restore the original potency of platelet extracts and therefore may modulate angiogenic effects of platelet-derived growth factors. Using cultured pulmonary artery VVEC as an angiogenic cell culture model, we investigated the effects of platelet extracts and adenine nucleotides on endothelial cell proliferation, migration and tube formation. Moreover, the angiogenic effects of platelet extracts and adenine nucleotides were validated in the mouse Matrigel plug assay. Our study revealed differences between angiogenic effects of platelet extracts and extracellular adenine nucleotides *in vitro *and *in vivo *and demonstrated that supplementation of platelet extracts with exogenous adenine nucleotides significantly potentiates morphogenetic effects of platelet extracts on Matrigel plug vascularization. These observations suggest that platelet-derived factors and extracellular nucleotides represent important regulatory signals for angiogenesis, and that delivery of entire platelet constituents may be considered as an efficient tool for therapeutic angiogenesis and wound healing applications.

## Methods

### Isolation of platelet extracts

Preserved human platelets were obtained from local Denver Metro Blood Banks the morning after the midnight expiration. The unit containing 200-225 ml was aliquoted into 6 × 50 ml tubes and then centrifuged at 2,500 × g for 15 min in a tabletop centrifuge (Sorvall Super T21, rotor ST-H750). After removal of the supernatant serum, the 6 pellets were resuspended in a 10-15 ml of 6 M urea in PBS and carried through 90 seconds of gentle sonication (Branson sonifier 450, duty cycle 20%, output control 2). The resulting solution was then centrifuged at 20,000 × g for 45 min to pellet out membrane fragments. The final supernatant solution was dialyzed against 10 mM HCl at 4°C using a 3.5 KDa cut-off membrane (five one liter exchanges each at least for 10 hours). After dialysis, protein concentration in platelet lysates was determined by the Bradford method and aliquots (15-30 ml) were stored at 4°C [19].

### Cultures of vasa vasorum endothelial cells

Pulmonary arteries were obtained from male Holstein calves that have been exposed to hypobaric hypoxia for two weeks (P_B _= 430 mmHg). Adventitia was dissected from the media, washed and enzymatically digested as previously described [21]. VVEC were purified from the co-cultures with adventitial fibroblasts using cloning rings and trypsinization techniques. Cells were maintained in DMEM media supplemented with 10% fetal bovine serum (FBS) and Endothelial Growth Supplement (Upstate Biotechnology, Charlottesville, VA). Isolated VVEC have been shown to exhibit endothelial morphology and express endothelial specific markers including PECAM, eNOS, and VEGFR [34]. All studies were performed on cells between passages 2 and 7. Under these conditions, cells sustained consistent functional, morphological, and phenotypical characteristics.

### DNA synthesis and cell proliferation assay

Cells were plated in 24 well plates at a density of 1.2 × 10^4 ^cells per well in DMEM supplemented with 10% FBS. On the next day cells were rinsed with phosphate-buffered saline (PBS), and incubated in DMEM without serum for 72 hrs. Cells were stimulated with platelet extracts (6.4, 16 and 64 μg/ml), extracellular ATP (100 μM), mixtures of MeSATP, ATPγS, MeSADP, and ADPβS (100 μM each), or combination of platelet extracts with adenine nucleotides in the presence of 0.125 μCi of [*methyl*-^3^H] thymidine (NEN Life Science Products, Boston, MA) for 24 hrs. Incorporated radioactivity was measured using a β-counter as previously described [21]. To determine the effects of extracellular nucleotides and platelet extracts on VVEC proliferation, cells were plated in 96 well plates at a density of 2.4 × 10^3 ^cells per well in DMEM supplemented with 1% FBS and grew for 48 and 60 hrs in the presence of platelet extracts (6.4, 16 and 64 μg/ml), extracellular ATP (100 μM), or their combination. Incubation media with all indicated components were changed daily, and cell proliferation rate was assessed using CyQuant proliferation kit (Invitrogen, Carlsbad, CA) according to manufacturer's protocol. Fluorescence intensity was determined using a plate reader (BMG LABTECH, Germany).

### Migration assay

Growth arrested VVEC (1.0 × 10^5 ^cells/well) were plated in 200 μl of serum free DMEM on permeable cell culture inserts (8.0 μM pore size, Costar Inc, Milpitas, CA) precoated with 1% gelatin (Sigma, St. Louis, MO). ATP (100 μM), platelet extracts (6.4-64 μg/ml) or a combination of both was added to the lower chamber containing 800 μl of serum free DMEM. After 24-hr incubation, cells remaining on the upper surface of the filter were wiped off, and migrated cells were fixed with methanol for 15 min and stained with 0.2% crystal violet in 2% (v/v) ethanol for a minimum of 15 min. Cells migrated through the filter were photographed under ×40 magnification of a phase contrast microscope in six random fields.

### *In vitro *Matrigel tube formation assay

Growth-arrested VVEC (1.25 × 10^5 ^cells/well in 24-well plate) were seeded on Growth Factor-Reduced Matrigel™ (BD Biosciences, San Jose, CA) in serum-free DMEM with or without ATP (100 μM), platelet extracts (16 or 32 μg/ml, as indicated in figure legends) or a combination of both. Cells were incubated for 10 hrs, and tube formation was analyzed using a phase contrast microscope at ×10 magnification. Photographs were taken using a digital camera connected to a Nikon microscope. Tube formation was analyzed by S.CORE Image Analysis (S.CO LifeScience) using three representative images for each experimental condition.

### Evaluation of purinergic receptor subtypes involved in VVEC angiogenesis

In DNA synthesis, migration and *in vitro *tube formation experiments, cells were pre-incubated with P2Y1 receptor antagonist, MRS2179 (2 μM), P2Y13 receptor antagonist, MRS2211 (25-50 μM, as indicated in figure legends); non-selective P2Y antagonist, suramin (25-50 μM, as indicated in figure legends), or non-selective P2Y/P2X antagonist, DIDS (25-50 μM, as indicated in figure legends) for 45 min prior to stimulation with extracellular ATP, platelet extracts, or a combination of ATP and platelet extracts.

### *In vivo *Matrigel plug assay

Aliquots (5-10 μl) of platelet extracts and/or adenine nucleotides were mixed with 200 μl of Growth Factor-Reduced Matrigel™ (BD Biosciences) on ice and were injected subcutaneously into the left and right lower abdominal side of 50 ICR mice (n = 4-5) using a 26-gauge needle. Control animals were injected with 200 μl of Matrigel containing 5-10 μl of vehicle (10 mM HCl). Each mouse was thoroughly anesthetized using gaseous isofluorane. After 7 days the animals were sacrificed by CO_2 _narcosis followed by cervical dislocation. The plugs and surrounding tissue were dissected from the abdomen and placed in 10% formalin at room temperature for two days. These were each dissected down the midline of the plug using a razor blade. The halves of each plug were placed along with the others from the same treatment into one of two tissue cassettes with the sectioned face down. This produced two "equivalent" slides of each group of 5 plug halves. The plugs were sectioned at 5-7 microns, stained with hematoxylin and eosin (H&E) and examined using a compound microscope at ×4-400 magnification. The tubes or capillaries visible at least 50 microns from a native tissue edge were considered to be newly created and not part of those preexisting in the native tissue. A total of 5-10 fields (×400 magnification) areas were chosen for quantification of tubular structures and functional capillaries. A capillary was defined as a ring or cylindrical structure with an internal endothelial cell lining (a lumen) in which red blood cells could be observed. Tubular forms (structures) were identical but lacked the presence of red blood cells. Furthermore, branched tubular forms were counted as one less than the number of arms (i.e. a y-shaped structure would be counted as 2, an x shaped structure as 3, etc) taking two of the arms, most closely aligned, to be forming one continuous tube. Representative images of Matrigel plugs were taken using an Olympus BX51 microscope at ×10 to ×40 magnification. To confirm the microvessel formation in matrigel plugs immunohistochemical staining with CD-31/PECAM -1 primary antibody (Dianova, Hamburg, Germany; 1:20 dilution) and secondary biotinylated goat anti-rat antibody (Santa Crus Biotechnology, CA; 1: 200 dilution) was performed according to the manufacturer's instructions. Staining was developed using Chromatogen AEC Single Solution (Invitrogen, Camarillo, CA). Slides were counterstained with Hematoxylin (Vector Labs, Burlingame, CA).

### Statistical analysis

For the analysis of variances between groups of data, one-way ANOVA was performed followed by Dunnett or Bonferroni test using GraphPad Prism 3.0 (GraphPad Software). Data are expressed as the means ± SE; *n *equals the number of replicates in one experiment or a number of observations in independent experiments. A p value of < 0.05 was considered statistically significant.

## Results

### Effect of platelet extracts and extracellular nucleotides on VVEC proliferation

**Figure 1:**
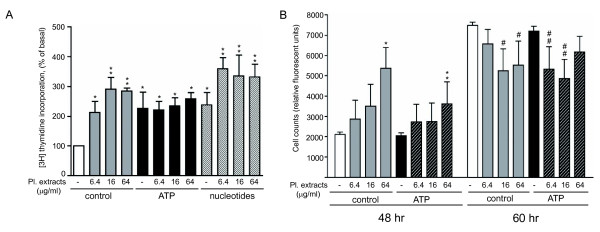
**Effect of extracellular nucleotides on platelet extract-induced mitogenic responses in VVEC**. **(A)**: Growth-arrested VVEC (72 hrs in serum-free DMEM) were stimulated with platelet extracts (6.4, 16 and 64 μg/ml) with or without ATP (100 μM) or a mixture of non-hydrolyzable nucleotide analogs (MeSATP, ATPγS, MeSADP, ADPβS, 100 μM each) in the presence of 0.125 μCi [^3^H]-thymidine for 24 hrs. Incorporated radioactivity was determined in total cell lysates as described in "Methods". Data represent the means ± SE from three independent experiments; * p < 0.05, ** p < 0.01 vs. nonstimulated control; **(B)**: VVEC were grown in DMEM/1% FBS in the presence of platelet extracts (6.4, 16, and 64 μg/ml), extracellular ATP (100 μM), or combination of both. Incubation media with all indicated components was changed daily. Cell proliferation rate was assessed using a fluorescent CyQuant proliferation kit (Invitrogen). Data represent the means ± SE from three independent experiments; * p < 0.05, ** p < 0.01 vs. nonstimulated control (48 hrs); # p < 0.05 vs. nonstimulated control (60 hrs); # # p < 0.05 vs. ATP-stimulated cells (60 hrs). No significant differences were observed between platelet-extract stimulated cells vs. platelet extract- and ATP-stimulated cells at 48 and 60 hrs (p > 0.05).

The effects of platelet-derived growth factors on VVEC mitogenesis were examined using [^3^H] thymidine incorporation and cell proliferation assays. Data in Figure 1A demonstrate that platelet extracts at the concentration of 6.4, 16 and 64 μg/ml significantly increased DNA synthesis in VVEC. Increased DNA synthesis was also observed when cells were stimulated with 100 μM ATP or a mixture of non-hydrolyzable nucleotides, ATPγS, ADPβS, MeSATP, and MeSADP (100 μM each). The effect of platelet extract was not significantly enhanced in the presence of ATP. However, it was potentiated in cells co-stimulated with platelet extracts (6.4 μg/ml) and the mixtures of nucleotide analogs.

Cell proliferation assay showed a gradual increase in cell numbers in response to stimulation with 6.4, 16 and 64 μg/ml of platelet extracts for 48 hrs (Figure 1B). The stimulatory effects of platelet extract were reduced to some extent in the presence of 100 μM ATP, although ATP itself did not have any effect on cell proliferation. After 60 hrs, a further increase in cell numbers was observed, however the proliferative responses in both control and platelet ATP-stimulated cells were partially attenuated in the presence of 16 and 64 μg/ml of platelet extracts and 6.4 and 16 μg/ml of platelet extract, respectively. At both 48 and 60 hrs, proliferative responses in platelet extract-stimulated cells remained without significant changes in the presence of 100 μM ATP. Together, these data demonstrate that in addition to DNA synthesis response induced by ATP and platelet extracts, more complex long-term regulatory signals generated by platelet extracts and extracellular ATP are necessary for endothelial cell growth.

### Effect of platelet extracts and extracellular nucleotides on VVEC migration

**Figure 2:**
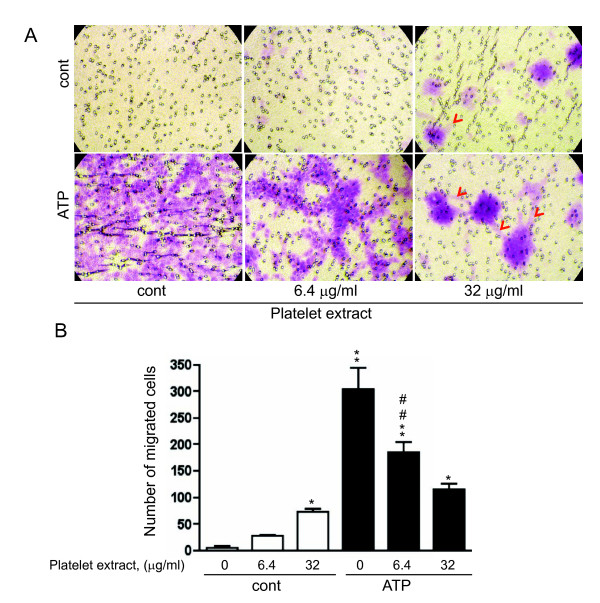
**Effect of extracellular nucleotides and platelet extract on VVEC migration**. Growth arrested VVEC (1.0 × 10^5 ^cells/well) were plated on top of inserts in serum free DMEM. Cell migration was stimulated by adding platelet extracts (6.4 and 32 μg/ml), ATP (100 μM) or their combination to the lower transwell compartment. **(A)**: Representative images of migrated cells; arrows indicate network-like structures **(B)**: Quantitative data represent the means ± SE from four independent experiments on three distinct VVEC populations; * p < 0.05, ** p < 0.01 vs. nonstimulated control; # # p < 0.01 platelet extract-stimulated cells vs. platelet extract- and ATP-stimulated cells.

Analysis of VVEC migration in the Boyden chamber assay demonstrated that platelet extracts at the concentration of 32 μg/ml increased the number of migrated cells. Noteworthy, clusters of migrated cells were formed on the underside of the transwell membrane (Figure 2A, red arrows). ATP had even greater chemotactic effect on VVEC. Although a number of migrated cells was gradually decreased when VVEC were co-stimulated with extracellular ATP and platelet extracts, the migration response remained statistically significant (compared to nonstimulated control). In addition, when cells were co-stimulated with 6.4 μg/ml of platelet extracts and ATP, the migration response remained statistically significant compared to the response induced by 6.4 μg/ml of platelet extracts alone. Figure 2B shows quantitative analysis of cell migration from several independent experiments. Surprisingly, we observed reproducible increases in cell clustering in response to co-stimulation with ATP and platelet extracts (6.4, and 32 μg/ml), suggesting that along with chemotaxis, platelet extracts exerted morphological rearrangements of endothelial cells.

### Effect of platelet extracts and nucleotides on VVEC tube formation

**Figure 3:**
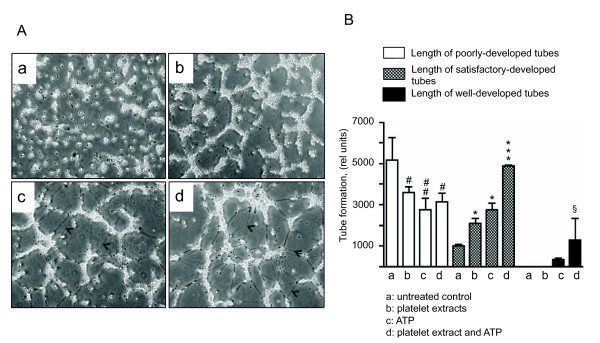
**Platelet extracts and extracellular ATP elicit VVEC rearrangements into tube-like network on Matrigel**. **(A)**: Growth-arrested VVEC (1.25 × 10^5 ^cells/well) were plated on growth factor-reduced Matrigel in serum free DMEM. Formation of tube-like networks was stimulated by the addition of platelet extracts (16 μg/ml), ATP (100 μM), or combination of both. After incubation, images were captured in three fields using Nikon microscope connected with digital camera. The data show one representative image for each experimental conditions; arrows indicate cells visible within the Matrigel; **(B)**: Quantitative evaluation of tube formation using S.CORE Image Analysis. Data represent the means ± SEM of three independent experiments; # p < 0.05, # # p < 0.05, vs. nonstimulated control (total length of poorly-developed tubes), * p < 0.05, *** p < 0.001 vs. nonstimulated control (total length of satisfactory-developed tubes), § p < 0.05 vs. nonstimulated control (total length of well-developed tubes).

To further evaluate potential morphogenetic effects of platelet extracts, we used a Matrigel tube formation assay *in vitro*. Incubation of quiescent VVEC on growth factor-reduced Matrigel for 10 hrs resulted in cell rearrangement into aligned and aggregated cell structures (poorly developed tubes), however no distinguishable network formation was observed (Figure 3A, *panel a*). Incubation of VVEC in the presence of platelet extracts (16 μg/ml) for 10 hrs resulted in visible cell rearrangements into tube-like networks (Figure 3A, *panel b*) evaluated as an increased total length of satisfactory-developed tubes and a decreased length of poorly-developed tubes (Figure 3B). The substantial tube formation effect was also observed in response to extracellular ATP (100 μM) with an increased length of satisfactory- and well-developed tubes and a decreased length of poorly-developed tubes (Figure 3A, *panel c *and Figure 3B). The combination of platelet extracts (16 μg/ml) and ATP (100 μM) resulted in cell rearrangement into a more distinguishable network pattern with a larger numbers of interconnected and well-developed tubes, indicating a potentiating effect of platelet extracts and ATP on VVEC morphogenesis (Figure 3A, *panel d *and Figure 3B).

### Involvement of purinergic receptor subtypes in ATP-stimulated angiogenic responses in VVEC

**Figure 4:**
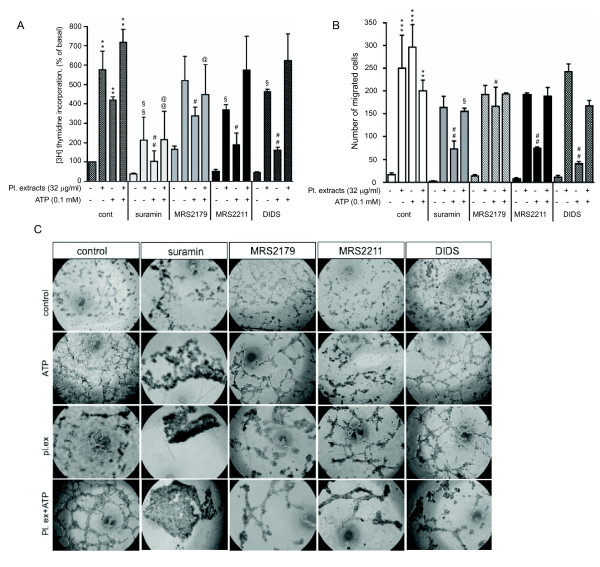
**Contribution of purinergic receptor subtypes in angiogenic responses to extracellular ATP in VVEC**. **(A)**: Growth-arrested cells were pre-incubated with purinergic receptor antagonists suramin (25 μM), MRS2179 (2 μM), MRS2211 (25 μM) or DIDS (25 μM) for 45 min or remained untreated and then stimulated with ATP (100 μM) in the presence of 0.125 μCi [^3^H]-thymidine for 24 hrs. Incorporated radioactivity was measured in total cell lysates using a β counter. Data represent means ± SE from four independent experiments conducted on two distinct VVEC populations; ** p < 0.01 vs. control; § p < 0.05, §§ p < 0.01 - platelet extract-stimulated cells vs. platelet extract-stimulated cells treated with antagonists; # p < 0.05, ## p < 0.01 ATP-stimulated cells vs. ATP-stimulated cells treated with antagonists; non-stimulated control; @ p < 0.05, @@ p < 0.01 ATP- and platelet extract-stimulated cells vs. ATP- and platelet extract-stimulated cells treated with antagonists; **(B) **Growth arrested cells were placed on top of inserts in serum free DMEM and pre-treated with suramin (50 μM), MRS2179 (2 μM), MRS2211 (50 μM) or DIDS (50 μM) for 45 min or remained untreated. Cell migration was stimulated by adding platelet extracts (32 μg/ml), ATP (100 μM) or their combination to the lower transwell compartment. Quantitative data represent the means ± SE from three independent experiments; ** p < 0.01, ***p < 0.001 vs. control; § p < 0.05 - platelet extract-stimulated cells vs. platelet extract-stimulated cells treated with antagonists; # p < 0.05, ## p < 0.01 ATP-stimulated cells vs. ATP-stimulated cells treated with antagonists; **(C) **Growth-arrested VVEC were plated on growth factor-reduced Matrigel in serum free DMEM and pre-treated with purinergic receptor antagonists as described above in the panel A. Tube formation was stimulated by the addition of platelet extracts (32 μg/ml), ATP (100 μM), or a combination of both. Data show images from one representative experiment. Similar results were obtained in at least three experiments conducted on distinct VVEC populations.

Our recent studies demonstrated that P2Y1 and P2Y13 purinergic receptors play a predominant role in Ca^2+ ^and mitogenic responses in VVEC [35]. To further characterize purinergic receptor subtypes in VVEC angiogenesis, we examined the effects of several purinergic receptor antagonists on VVEC DNA synthesis, migration and tube formation. Pre-treatment of VVEC with suramin (non-selective P2Y receptor antagonist), MRS2179 (P2Y1 selective receptor antagonist), MRS2211 (P2Y13 selective receptor antagonist), and DIDS (P2Y/P2X non-selective receptor antagonist) significantly inhibited DNA synthesis in response to ATP (Figure 4A). Pre-treatment with suramin, MRS2179, but not MRS2211 and DIDS significantly reduced DNA synthesis in response to a combination of ATP and platelet extracts. Surprisingly, pre-treatment with suramin, MRS2211, and DIDS also reduced DNA synthesis response to platelet-extract, suggesting that endogenous purinergic receptor ligands from these mixtures contribute to VVEC mitogenesis. In addition, according to some reports, the inhibitory effect of suramin can be due to the interference of growth factor-receptor binding [36].

We also found that suramin, MRS2179, MRS2211, and DIDS all significantly reduced VVEC migration in response to ATP, but only modestly, in response to stimulation with ATP in a combination with platelet extracts (Figure 4B). Potent inhibitory effects of suramin and DIDS on VVEC migration may indicate that in addition to P2Y13 and P2Y1, additional subtypes of P2Y and P2X receptors contribute to nucleotide-induced VVEC migration.

The effects of purinergic receptor antagonists were further evaluated in the *in vitro *tube formation assay. Representative images for each condition are shown in Figure 4C. Pre-treatment of VVEC with suramin had a potent inhibitory effect on VVEC tube formation under each condition tested. Pre-treatment of VVEC with MRS2179 decreased tube formation in response to ATP, platelet extracts, and combination of ATP with platelet extracts. Pre-treatment with MRS2211 decreased tube formation in response to ATP and the combination of ATP with platelet extracts, whereas tube formation in response to platelet extracts was less affected. Pre-treatment with DIDS had a small effect on ATP-induced tube formation, modest effects on platelet extract- induced tube formation, and potent effect on tube formation stimulated by combination of ATP and platelet extracts. Together, these data show a differential contribution of P2 receptors in VVEC morphogenesis. The effects are mediated presumably by P2Y1, P2Y13 and possibly, P2Y2 receptors. The role of P2X receptors requires more specific evaluation using selective agonists and antagonists.

### Effect of platelet extracts and nucleotides on Matrigel plug neovascularization *in vivo*

**Figure 5:**
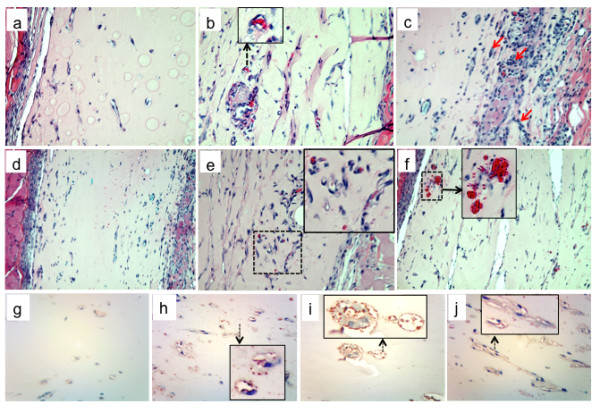
**The effects of platelet extracts and adenine nucleotides on Matrigel plug vascularization**. Matrigel (200 μl per plug) was mixed with platelet extracts (29 μg/ml and 58 μg/ml), ATP (1 mM), mixture of MeSATP, ATPγS, MeSADP, and ADPβS (0.1 mM, 0.5 mM or 1 mM of each), combination of nucleotides and platelet extracts (concentrations as indicated), or vechicle (10 mM HCl in PBS) and injected subcutaneously into ICR mice (n = 4-5 for each group). After 7 days, mice were sacrificed, Matrigel plugs were excised, and 5-7 μm-thick sections were prepared from formalin fixed plugs/paraffin-embedded plugs. H&E staining was performed for identification of plug cellularity and tube formation. Representative images show (*a*): control plugs, ×40; *(b)*: plugs containing 58 μg/ml platelet extract (×40; insert shows magnified view of an capillary containing red blood cells); *(c-d)*: plugs containing 0.5 mM purine nucleotides (*panel c *= ×40, *panel d = *x10 magnification; arrows on the *panel c *indicate areas of increased plug cellularity); platelet extracts (58 μg/ml), mixture of MeSATP, ATPγS, MeSADP, and ADPβS (0.1 mM, 0.5 mM or 1 mM of each), *(e-f)*: plugs containing platelet extracts (58 μg/ml) and mixture of nucleotides (0.5 mM of each); *panel e*: plug with increased amount of functional capillaries (enlarged in the insert); *panel f*: blood cell infiltrates around new formed capillaries (enlarged in the insert); *(g-j)*: Identification of PECAM-positive cells in control plugs *(g) *and plugs containing 58 μg/ml platelet extract *(h)*, mixture of nucleotides (0.5 mM of each), and combination of 58 μg/ml platelet extract and mixture of nucleotides (0.5 mM of each, *j*). PECAM-positive cells are shown enlarged in the inserts.

**Figure 6:**
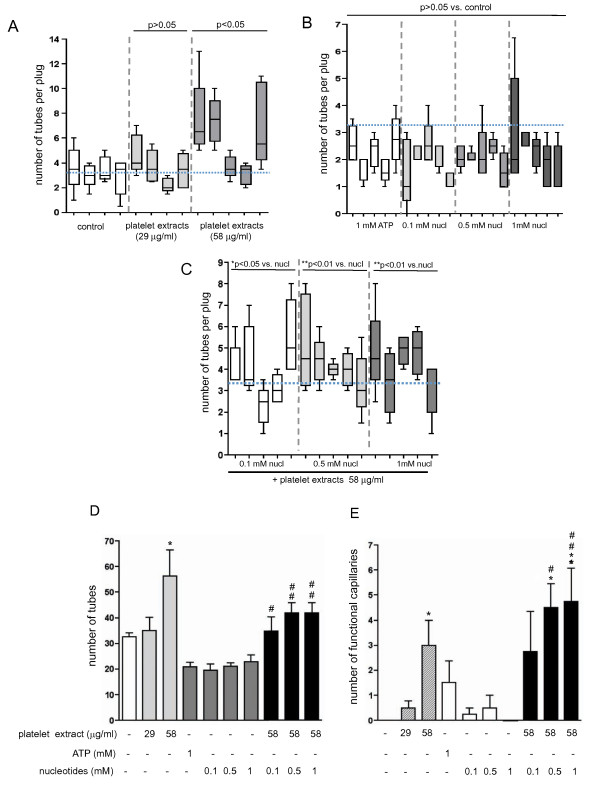
**Quantitative analysis of tube formation in nucleotide-containing Matrigel plugs**. Data in panels A-C represent means ± SE for the amount of tubes detected in right- and left-side placed plugs for each animal in the group. Five to ten random fields were analyzed per each section. **(A)**: Tube formation in control and platelet extract containing Matrigel plugs (concentrations as indicated). Total number of tubes per animal group were use for statistical analysis of variances; p > 0.05 and p < 0.05 vs. control group; **(B)**: Tube formation in Matrigel plugs containing ATP or mixtures of MeSATP, ATPγS, MeSADP, and ADPβS (concentrations as indicated). Total number of tubes per animal group were use for statistical analysis of variances; p > 0.05 vs. control group, shown in panel A); **(C)**: Tube formation in Matrigel plugs containing platelet extracts and mixtures of MeSATP, ATPγS, MeSADP, and ADPβS (concentrations as indicated); *p < 0.05 vs. nucleotides alone, **p < 0.01 vs. nucleotides alone); **(D)**: Summary of the analysis of tube formation for all experimental conditions. Data are mean ± SE for the total amount of tubes for each group; *p < 0.05 vs. control; # p < 0.05 vs. nucleotides alone; # # p < 0.01 vs. nucleotides alone; **(E)**: Quantitative analysis of functional capillaries in Matrigel plugs. The capillaries of at least 50 microns from a native tissue edge were considered newly created within the plug and were not part of those preexistent in the native tissue. Five to ten random fields were analyzed per each section. Data are means ± SE (n = 5) for each condition; *p < 0.05 vs. control; **p < 0.01 vs. control # p < 0.05 vs. nucleotides alone; # # p < 0.01 vs. nucleotides alone.

Considering the observed morphogenetic effects of platelet extracts and extracellular ATP on VVEC, we next examined the effect of these angiogenic modulators using the *in vivo *Matrigel plug assay. Subcutaneous injection of 200 μl of growth factor-reduced Matrigel in 50 ICR mice did not induce neovessel formation in the Matrigel plugs above that typically seen with Matrigel alone (Figure 5, *panel a*; Figure 6A). Injection of Matrigel supplemented with 58 μg/ml platelet extracts gave an increased number of tubes per plug, changing from 3.2 observed in the control (n = 4) to 7.5 (n = 3); (Figure 5, *panel b*; Figure 6A). The effect of 29 μg/ml platelet extract on tube formation *in vivo *was not statistically significant (Figure 6B).

Furthermore, the effects of adenine nucleotides on Matrigel plugs vascularization were evaluated. Data in Figure 5 show that injection of Matrigel supplemented with either ATP (1 mM) or mixtures of non-hydrolyzable nucleotides, ATPγS, ADPβS, MeSATP, MeSADP (0.1, 0.5 and 1 mM of each) did not result in increased Matrigel plug vascularization (Figure 5*panels c, d*). The number of tubes per plug was lower than in the control group (Figure 6B). Notably, a considerable variability in Matrigel vascularization was observed between individual animals within the group. Moreover, visible infiltration of inflammatory cells was observed in ATP supplemented Matrigel plug retrieved from one animal (Figure 5, *panel d*).

Since ATP potentiated the effect of platelet extract on VVEC tube formation *in vitro*, it was expected that a similar effect would be observed in plug vascularization *in vivo*. Data in Figure 5, *panel e *show that subcutaneous injection of Matrigel supplemented with mixtures of non-hydrolyzable nucleotides, ATPγS, ADPβ, MeSATP, and MeSADP (each 0.1, 0.5 or 1 mM) and platelet extracts (58 μg/ml) resulted in the enhanced formation of tubular structures and functional capillaries in Matrigel plugs. In addition, in some plugs, blood infiltrates can be observed, suggesting that some neovessels/immature blood conduits, prone to rupture, might have formed there (Figure 5, *panel f*). The tube numbers in nucleotide -containing plugs were almost the same for each nucleotide concentration, and these numbers were lower than those in the plugs supplemented with platelet extracts only (Figure 6C). Data in figure 6D summarize the results of Matrigel plug vascularization for all experimental groups. A statistically significant increase in number of tubes was observed in Matrigel plugs containing 58 μg/ml platelet extracts and 58 μg/ml in combination with nucleotide mixtures. Remarkably, the combination of platelet extracts and nucleotides also gradually increased the number of functional capillaries; the most dramatic effect was observed in Matrigel plugs, supplemented with 0.5 and 1 mM of nucleotides (Figure 6E). Histological evaluation of Matrigel plugs suggested that both immature tubular structures and functional capillaries could be expected in Matrigel plugs. To confirm the microvessel formation, plugs were immunostained for CD31/PECAM-1. The number of PECAM-1-positive endothelial cells was higher in Matrigel plug supplemented with platelet extracts, ATP, and mixtures of nucleotide analogs compared to control plugs (Figure 5C, *panels g-j*).

Altogether, the results from our *in vitro *and *in vivo *studies suggest that platelet extract and adenine nucleotides promote distinct but likely complementary signaling events, leading to functional neovessel formation.

## Discussion

Development of improved strategies for therapeutic angiogenesis remains an imperative task for treatment of a variety of pathological conditions. Of all the circulating blood cells, platelets are the most abundant sources of endogenous angiogenic regulators. In this study we challenged the hypothesis that combination of platelet-derived factors with extracellular nucleotides, also known as regulators of the vascular growth, would have even more potent angiogenic effects *in vitro *and *in vivo*. Our data demonstrated that platelet extracts and extracellular adenine nucleotides exerted similar, but not identical effects on endothelial proliferation, migration and tube formation. The main conclusion from this study is that extracellular adenine nucleotides modulate angiogenic effects of platelet extracts *in vitro *and, importantly, lead to a greater amount of functional capillary formation in Matrigel plugs *in vivo*.

As mentioned above, platelets contain multiple constituents including angiogenic mediators, extracellular matrix components, nucleotides, sugars, growth factors, antimicrobial peptides, and cytokines that can support wound healing and angiogenesis [6-8,12,14,30,33]. Angiogenic effects of platelets have been previously demonstrated in several model systems. Recent reports have shown some examples that platelet rich plasma (PRP) and platelets can substitute for animal serum as a nutrient source for human cells in culture and therefore, may have the capacity to provide nourishment or survival factors to cells at risk from the compromised vascular supply at a wound site [[37,38] and Mark Roedersheimer, unpublished observation]. It was demonstrated, that a potent chemotactic and angiogenic activity is generated by factors released from activated platelets [7,9,30]. The presence of platelets not only stimulates vessel growth but also plays a critical role in preventing hemorrhage from the angiogenic vessels [11] supporting a theory of functional link between hemostasis and angiogenesis.

However, despite a strong evidence of angiogenic effects of platelets, there is also some degree of skepticism about the utility of platelet-derived components for a modulation of angiogenesis due to a high degree of variability in clinical outcomes, which are probably associated with: (i) lack of sufficient release of key growth factors following an incomplete platelet "activation" with a single factor, such as thrombin, during PRP preparation; (ii) failure to separate the platelet-derived growth factors from the bulk of the highly concentrated serum components, which may limit bioavailability; (iii) failure to determine at least the protein concentration of the active platelet extract (separated from the plasma) and using this to establish a proper dosing; and iv) variability in the composition, and therefore activity, of platelet extracts from different individuals that may need to be accounted for in the dosing. Reducing variability in the clinical response to PRP remain as important goal in bringing platelet extract preparation methods to full utility.

In the present study we demonstrated that total cell extracts isolated from preserved platelets have an angiogenic potential. Our approach of using total platelet extracts assumed that a crude extract isolated by a lysis of platelet pellet, even though lacking the ability of intact platelets to sense and respond to signals in a tissue environment, can still be used to produce a balanced regenerative response in a tissue. According to our established procedure, soluble platelet extracts were prepared by platelet sonication and subsequent stabilization of soluble components through the dialysis of cellular extract against 10 mM HCl in a 3.5 KDa cut-off membrane. Based on some reports, mildly acidic environment (also typical for wound sites) may possibly optimize the stability and activity of platelet-derived factors such as PDGF and TGFβ [39,40]. However, the dialysis step removes small molecular weight soluble components, including nucleotides and amino acids from the fraction of growth factors and cytokines. Therefore, it can be assumed that when platelet extract preparation includes a dialysis step, supplementation (or "reconstruction") of dialyzed platelet extracts with exogenous adenine nucleotides could modulate or potentially enhance the angiogenic effects of platelet-derived extracts.

A variety of pathological conditions in the vasculature is accompanied with elevated levels of extracellular ATP and ADP, platelet immobilization, activation, and their adhesion to endothelial cells, with ultimate endothelial cell activation. These events may possibly lead to angiogenesis and initiation of vascular regeneration processes [23,41-44]. Activated platelets release factors capable of promoting hematopoietic stem cell migration into a vascular injury site and their differentiation into endothelial cells, which may favor survival via angiogenic signaling pathways [32,45]. We expect that extracellular nucleotides released from platelets and endothelial cells could act in concert with multiple growth factors and cytokines. Previously, we demonstrated that extracellular ATP exerts dramatic effects on VVEC mitogenesis, migration and tube formation [21,23]. However, angiogenic effects of extracellular nucleotides in the *in vivo *models of angiogenesis, as well as combined effects of extracellular nucleotides and platelet-derived growth factors on angiogenesis have not yet been investigated. Our present study provides new evidence that platelet-derived extracts and adenine nucleotides exerted diverse, but complementary angiogenic effects in isolated VVEC. Platelet extract induced DNA synthesis and affected proliferative responses in VVEC. The proliferative response was at its highest at 48 hrs, followed by a decrease at 60 hrs. This biphasic effect may suggest a possibility that the initial increase in VVEC proliferation may be followed by more long-term regulatory signals leading to endothelial differentiation, stabilization and/or morphological changes. Strikingly, extracellular ATP, despite strong effects on DNA synthesis, did not induce VVEC proliferation and to some extent diminished proliferative responses induced by platelet extracts measured at 48 hrs, again, suggesting prolonged and more complex integrative signaling events underlying the action of extracellular nucleotides and platelet-derived angiogenic factors.

Our study also revealed differences in extracellular ATP- and platelet extract-induced migratory responses. Extracellular ATP had a more potent effect (~75- fold) on VVEC migration in contrast to the effect induced by platelet extracts (~7-20-fold). However, more dramatic effects (up to 65-fold) can be observed in response to platelet extracts derived some units of preserved human platelets (Figure 4B and unpublished observations). Interestingly, in response to extracellular ATP all migrated VVEC adhered to and spread uniformly on the underside of the transwell chamber membrane, whereas in response to platelet extracts, aggregates of migrated cells were observed on the underside of the membrane. We also found that platelet extracts, in a concentration-dependent manner, diminish ATP-induced VVEC migration, which was accompanied by a more dramatic cell clustering on the underside of the chamber membrane. Similar to our findings, a recent study by O'Connor demonstrated "adherent migration" of THP-1 in response to platelet soluble fractions [33], suggesting that even in the migration assay setting, platelet extracts induce structural and adhesive changes in endothelial cells. This morphogenetic effect of platelet extract was further demonstrated in the Matrigel tube formation assay. We found that platelet extract and extracellular ATP were almost equipotent in the induction of satisfactory-developed tubes on growth factor-reduced Matrigel. In addition, the total number of well-developed tubes was significantly increased when VVEC were co-stimulated with platelet extracts and extracellular ATP. Together, the *in vitro *studies demonstrated that both extracellular ATP and platelet extracts exert angiogenic effects in VVEC, but only morphogenetic effect was potentiated in response to the combined action of extracellular ATP and platelet extracts. Further investigation is necessary to define molecular mechanisms contributing to intracellular signaling cross-talk initiated by purinergic receptors and receptor tyrosine kinases in VVEC.

Recenty, we demonstrated a predominant involvement of P2Y1 and P2Y13 receptors in Ca^2+ ^and mitogenic response in VVEC [35]. In this study we further investigated a contribution of purinergic receptor subtypes in VVEC angiogenesis. Using an antagonist approach, we demonstrated an involvement of P2Y1 and P2Y13 receptors in ATP-induced DNA synthesis and tube formation, as well as an involvement of P2Y13 receptors in VVEC migration. An inhibitory effects of suramin (non-selective P2Y antagonist) on ATP-induced DNA synthesis and tube formation may also suggest a contribution of P2Y2 receptors in these angiogenic responses. In addition, potent inhibitory effects of DIDS on DNA synthesis, migration, and, to a lower extent, tube formation may suggest an involvement of P2X receptors in mediating nucleotide signaling in VVEC. We also found, that some angiogenic effects of platelet extract, although to a different degree, can be attenuated by purinergic receptor antagonists (suramin and MRS2179). Whether this can be explained by the presence of residual endogenous nucleotides or other purinergic receptor ligands (such as cysteinyl leukotrienes) in platelet extracts remains to be determined and represents an interesting possibility for future investigations.

Consistent with the results from the *in vitro *studies, platelet-derived extracts displayed a dose-dependent angiogenic response in the Matrigel plug assay in 50 ICR mice. The effect was evaluated in terms of the number of tube-like structures, as well as functional capillaries containing red blood cells sufficiently far from a native tissue boundary to exclude their preexistence. We have previously shown that even very low concentrations of platelet-derived extracts (<50 μg/ml) speeded up the healing process of incision wounds placed on the backs of mice (unpublished observation). However, some variations in minimal effective concentration of platelet extracts were observed among human plasma sources. For instance, platelet extracts used in this study exhibit the highest activity at concentrations between 16 and 64 μg/ml. The functionality of capillaries in Matrigel plugs was evaluated based on microscopic detection of erythrocytes within the capillary lumen, suggesting a link to the systemic circulation. In agreement with the *in vitro *data, we observed increased formation of tube-like structures in Matrigel plugs supplemented with platelet extract. Despite a number of studies showing angiogenic effects of extracellular nucleotides in various cell systems, the angiogenic effects of extracellular nucleotides in the *in vivo *Matrigel plug model have not been previously investigated. To our surprise, tube formation in the Matrigel plugs supplemented with extracellular ATP was even lower than the tube formation in the control plugs.

It would have been expected that extracellular ATP might be hydrolyzed to adenosine by endothelial and blood cell ecto-nucleotidases, resulting in decreased plug vascularization. Therefore, we evaluated the effect non-hydrolyzable nucleotide analogs, ATPγS, ADPβS, MeSATP, MeSADP. No additional tube formation was observed in response to these nucleotides, suggesting that decreased plug vascularization in response to ATP could not be explained by its hydrolysis to adenosine. Remarkably, we found more cellularity in some Matrigel plugs, containing mixtures of high concentrations of extracellular nucleotides (1 mM). Considering that monocytes/macrophages and endothelial progenitor cells may contribute to neovascularization, and can be attracted by platelet-derived microparticle components [32,46-49], we speculate that inflammatory and/or progenitor cells may also contribute to angiogenic responses in Matrigel plugs. This possibility will be further investigated in our laboratory. Histological evaluation of Matrigel plugs revealed that both immature tubular structures and functional capillaries were formed. Although tube formation in the Matrigel plugs, supplemented with extracellular ATP was even lower than the tube formation in the control plugs, higher numbers of functional capillaries and PECAM -positive cells were observed in ATP and nucleotide-containing Matrigel plugs compared to control. In some plugs, blood infiltrates were found in addition to well-formed capillary structures, indicating that newly-formed vessels could be, at least in part, immature and leaky. In the meantime, the amount of functional capillaries and PECAM-positive cells was even higher in Matrigel plugs containing platelet extracts and nucleotides (compared to Matrigel plugs containing either nucleotide or platelet extract). These observations are consistent with the idea that extracellular nucleotides and platelet -derived growth factors provide complementary regulatory signals necessary to promote neovessel growth and maturation.

## Conclusions

In conclusion, our study provides new evidence for the angiogenic effects of platelet-derived extracts in combination with extracellular adenine nucleotides. These results shed new light on the role of platelets and extracellular nucleotides in the regulation of vascular hemostasis and angiogenesis. Our findings could be clinically applicable for the optimization of wound healing, tissue engineering or vessel stabilization, important in many pathological conditions, such as those observed in tumor and diabetes complications.

## Competing interests

The authors declare that they have no competing interests.

## Authors' contributions

MR performed Matrigel plug experiments in mice and analyzed Matrigel plug vascularization. HN performed angiogenic assays and analyzed the data. NB isolated VVEC and performed immunohistochemistry. AAS performed migration data analysis. KRS provided lung tissue for VVEC isolation and critical comments for the manuscript. EVG designed the study, performed in vitro experiments and analyzed the data. The manuscript was written by EVG and MR. All authors read and approved the final manuscript.
